# Distinct Epidermal Keratinocytes Respond to Extremely Low-Frequency Electromagnetic Fields Differently

**DOI:** 10.1371/journal.pone.0113424

**Published:** 2014-11-19

**Authors:** Chao-Ying Huang, Chun-Yu Chuang, Wun-Yi Shu, Cheng-Wei Chang, Chaang-Ray Chen, Tai-Ching Fan, Ian C. Hsu

**Affiliations:** 1 Department of Biomedical Engineering and Environmental Sciences, National Tsing Hua University, Hsinchu, Taiwan; 2 Institute of Statistics, National Tsing Hua University, Hsinchu, Taiwan; 3 Magnet Group, Instrumentation Development Division, National Synchrotron Radiation Research Center, Hsinchu, Taiwan; National Research Council, Italy

## Abstract

Following an increase in the use of electric appliances that can generate 50 or 60 Hz electromagnetic fields, concerns have intensified regarding the biological effects of extremely low-frequency electromagnetic fields (ELF-EMFs) on human health. Previous epidemiological studies have suggested the carcinogenic potential of environmental exposure to ELF-EMFs, specifically at 50 or 60 Hz. However, the biological mechanism facilitating the effects of ELF-EMFs remains unclear. Cellular studies have yielded inconsistent results regarding the biological effects of ELF-EMFs. The inconsistent results might have been due to diverse cell types. In our previous study, we indicated that 1.5 mT, 60 Hz ELF-EMFs will cause G1 arrest through the activation of the ATM-Chk2-p21 pathway in human keratinocyte HaCaT cells. The aim of the current study was to investigate whether ELF-EMFs cause similar effects in a distinct epidermal keratinocyte, primary normal human epidermal keratinocytes (NHEK), by using the same ELF-EMF exposure system and experimental design. We observed that ELF-EMFs exerted no effects on cell growth, cell proliferation, cell cycle distribution, and the activation of ATM signaling pathway in NHEK cells. We demonstrated that the 2 epidermal keratinocytes responded to ELF-EMFs differently. To further validate this finding, we simultaneously exposed the NHEK and HaCaT cells to ELF-EMFs in the same incubator for 168 h and observed the cell growths. The simultaneous exposure of the two cell types results showed that the NHEK and HaCaT cells exhibited distinct responses to ELF-EMFs. Thus, we confirmed that the biological effects of ELF-EMFs in epidermal keratinocytes are cell type specific. Our findings may partially explain the inconsistent results of previous studies when comparing results across various experimental models.

## Introduction

Because of the increased usage of electric appliances that can generate 50 or 60 Hz electromagnetic fields, people have been increasingly exposed to extremely low-frequency electromagnetic fields (ELF-EMFs). In the past 3 decades, a worldwide debate has focused on the biological effects of ELF-EMFs on human health. According to the guidelines recommended by the International Commission on Non-Ionizing Radiation Protection (ICNIRP) in 2010 [1], exposure to magnetic flux density from power lines (50 or 60 Hz) must be less than 0.2 mT and 1.0 mT for general public and occupational exposure, respectively. However, the biological mechanism facilitating the effects of ELF-EMFs remains unclear. Cellular studies have yielded inconsistent results regarding the biological effects of ELF-EMFs.

Some scientists have stated that ELF-EMFs can promote cell proliferation [2]–[4], whereas others have indicated that ELF-EMFs can inhibits cell proliferation [5]–[8]. However, certain studies have indicated that no effect of ELF-EMFs was exerted on either cell growth or cell proliferation [9], [10]. The inconsistent results have been suggested as being due to distinct cell types [11], [12]. In our previous study [13], we observed that 1.5 mT, 60 Hz ELF-EMFs inhibit cell growth and cause G1 arrest in HaCaT cells, which is a human keratinocyte cell line that contains inactive mutant p53 [14]. In this study, we investigated whether ELF-EMFs cause similar effects in different epidermal keratinocytes under the same exposure conditions when using the same experimental design. We used primary normal human epidermal keratinocytes (NHEK) as experiment model. Our results revealed that ELF-EMF exposure exerted no effects on cell growth, cell proliferation, and cell cycle distribution of NHEK cells. All of these observations contradict the results regarding HaCaT cells. The results indicated that the 2 epidermal keratinocytes responded to ELF-EMFs differently. This finding confirms the suggestion of a previous report [12]; in other words, the contradictory biological effects induced by ELF-EMFs might be due to differences in the cell types applied.

## Materials and Methods

### Cell culture

Primary NHEK cells from neonatal foreskin (PCS-200-010) were purchased from American Type Culture Collection (ATCC, Manassas, VA). The NHEK cells were cultured in the Dermal Cell Basal Medium (ATCC, Manassas, VA) supplemented with the Keratinocyte Growth Kit (ATCC, Manassas, VA) and antibiotics (10 µg/mL of gentamicin, 0.25 µg/mL of amphotericin B, 10 U/mL of penicillin, 10 µg/mL of streptomycin, 25 ng/mL of amphotericin B; ATCC, Manassas, VA) according to the manufacturer's instructions. Immortalized nontumorigenic human keratinocytes, HaCaT cells [15], were kindly provided by Dr. Norbert E. Fusenig (German Cancer Research Center, Heidelberg, Germany). The HaCaT cells were cultured in Dulbecco's modified Eagle's medium (Gibco BRL, Grand Island, NY) supplemented with 10% heat-inactivated fetal bovine serum (HyClone, Laboratories Inc., Logan, UT) and antibiotics (100 U/mL of penicillin, 100 µg/mL of streptomycin, and 2 mM L-glutamine; Gibco BRL, Grand Island, NY). Both the NHEK and HaCaT cells were incubated at 37°C in humidified air containing 5% CO_2_.

### ELF-EMF exposure system and UVB irradiation

The ELF-EMF exposure system and its quality control were described in our previous study [13]. Briefly, all sham-exposed cells were cultured in a chamber magnetically shielded using a mu-metal (Aircraft Materials UK, Princes Risborough, Buckinghamshire, UK) box in the same incubator in which the exposed cells were incubated. When the coil system generated the uniform 1.5 mT ELF-EMF (the exposed environment), the intensity of the ELF-EMFs within the mu-metal box was 1.50±0.03 µT (the unexposed environment). The temperature in the incubator was monitored using a thermometer (TES, Taipei, Taiwan) in the unexposed (36.9±0.3°C) and exposed (36.9±0.3°C) environments. The pH value of the culture medium was measured using a Corning pH meter 320 (Corning, NY), and the pH values was 7.33±0.02 for the culture medium in the unexposed environment and 7.34±0.02 for the culture medium in the exposed environment.

A 6-W UVB fluorescent tube (Spectroline EB-160C, Spectronics Corporation, Westbury, NY) was used as the UVB light source. The UVB dose was measured using a photometer (model IL 1400A, International Light Inc., Newburyport, MA). The positive controls were collected 24–144 h after being subjected to 21.5 J/m^2^ of UVB irradiation. The detail of UVB irradiation procedures as well as the quality control of illumination fields have been described in our previous studies [16], [17].

### Cell growth curves of 2 epidermal keratinocytes

NHEK cells (1.0−4.5×10^4^) were seeded in a 10 cm tissue culture dish (BD Biosciences, San Jose, CA) and incubated for 24 h prior to exposure to an ELF-EMF. NHEK cells were exposed to a 1.5 mT, 60 Hz field in the Helmholtz coil system for all experimental periods. The sham-exposed cells were cultured in the same incubator but shielded by the mu-metal. At each time point in the experiments, nonviable cells were identified and excluded using trypan blue (Sigma, St. Louis, MO) dye exclusion counting by employing a hemocytometer. HaCaT cells (1.0−1.5×10^5^) were seeded in a 10 cm tissue culture dish (BD Biosciences, San Jose, CA) and incubated for 12 h prior to exposure to an ELF-EMF for a simultaneous exposure experiment involving the NHEK and HaCaT cells. A minimum of 3 independent experiments were conducted.

### Colony formation assay of 2 epidermal keratinocytes

Colony formation assays can provide cell proliferation information complement to that of cell growth curve. The cell growth and proliferation ability were analyzed by cell growth curves and colony forming assay, respectively [18]. The procedure for the colony assay was performed as previously described [13]. In brief, both sham and exposed NHEK (2.0×10^3^) and HaCaT (5×10^2^) cells were seeded in a 10 cm petri dish and then placed in the incubator. After 144 h of ELF-EMF exposure, the colonies of each petri dish were washed with PBS and fixed with 3 mL of Carnoy's solution (methanol: acetic acid 3∶1, v/v) for 3 min. Then cells were washed with PBS and fixed in 100% methanol for 30 min; subsequently, they were stained with the KaryoMAX Giemsa Stain Solution (Gibco BRL, Grand Island, NY) for 10 min. Each dish was washed with water and dried overnight at room temperature. A colony was defined as a cluster of more than 50 cells. Three independent experiments were conducted.

### Cell cycle analysis using propidium iodide staining

NHEK cells were harvested and fixed with 6 mL of prechilled 83% ethanol/PBS at −20°C overnight. The fixed cells were centrifuged at 1500 rpm for 5 min and washed twice with ice-cold PBS. The resulting pellet was resuspended in 20 µg/mL of a propidium iodide (PI; Molecular Probes Inc., Eugene, OR) solution with 200 µg/mL of RNase A (Invitrogen, Carlsbad, CA) and 0.02% Triton X-100 (Sigma, St. Louis, MO), and then incubated in the dark at 37°C for 30 min. DNA content was subsequently analyzed by conducting FACSCanto flow cytometry (Becton Dickinson, San Jose, CA). Each cell cycle phase was analyzed using ModFit LT software (Verity Software House, Topsham, ME). Three independent experiments were conducted.

### Quantitative real-time polymerase chain reaction

Total RNA was extracted from NHEK cells using TRIzol reagent (Invitrogen, Carlsbad, CA). A quantitative real-time polymerase chain reaction (qRT-PCR) assay was performed using a Power SYBR Green Master Mix (Applied Biosystems, Warrington, UK) and an ABI Prism 7300 Real-Time PCR System (Applied Biosystems, Foster City, CA). Gene-specific primers were designed using Primer-BLAST [19]. All primers used in this study are listed in Table S1. The PCR reaction was performed under the following conditions: 40 cycles of denaturing (95°C, 15 s), annealing (60°C, 30 s), and extension (72°C, 45 s) processes. *GAPDH* was used as a reference gene in the qRT-PCR experiments for normalization in each sample. The experiments were performed in triplicate to confirm reproducibility.

### Immunoblotting assay

Cell lysates were prepared and western blots were performed as previously described [13]. We performed 2 parallel samples in an independent experiment under ELF-EMF exposure, and the cell lysates of 2 parallel samples were pooled into a vial to perform the immunoblotting. Three independent experiments were performed. The primary antibodies used for western blotting were: ATM (2873), Chk2 (3440), phospho-Chk2 Thr68 (2661), p53 (2527) and phospho-p53 Ser15 (9286) from Cell Signaling Technology (Beverly, MA); phospho-ATM Ser1981 (2152-1) from Epitomics (Burlingame, CA); and β-Actin (sc-47778) from Santa Cruz (Santa Cruz, CA). The horseradish peroxidase conjugated secondary antibodies were: anti-mouse IgG HRP-linked antibody (7076) and anti-rabbit IgG HRP-linked antibody (7074) from Cell Signaling Technology (Beverly, MA).

### Statistical analysis

The data, which are presented as the mean ± SD, were obtained after conducting at least 3 independent experiments and subsequently analyzed using Prism 5 software (GraphPad Software, San Diego, CA, USA). The qRT-PCR data (shown in Table 1) are presented as the fold change in gene expression normalized to *GAPDH* (used as a reference gene) and relative to the unexposed controls using the 2^−ΔΔC_T_^ method [20]. Differences between the sham and exposed groups were analyzed using the Student's *t*-test and considered statistically significant at *P*<0.05.

**Table 1:** The relative gene expression levels of the 12 cell cycle-related genes of the exposed NHEK cells relative to that of the unexposed controls.

	Exposure Time		
Gene Name	96 h	120 h	144 h
**GADD45A**	0.82–1.13	0.62–1.14	0.88–1.13
			
**CDKN1A**	0.95–1.26	0.80–1.25	0.84–1.07
			
**CCNA2**	0.93–1.15	0.76–1.27	0.85–1.10
			
**CCNB1**	1.00–1.05	0.84–1.33	0.86–1.09
			
**CCND2**	0.96–1.17	0.84–1.23	0.78–1.05
			
**CCNE1**	0.96–1.12	0.85–1.08	0.96–1.35
			
**CDK1**	0.89–1.10	0.81–1.30	0.91–1.18
			
**CDK2**	0.86–1.19	0.49–0.96	0.97–1.47
			
**CDK4**	0.76–1.02	0.83–1.07	0.82–1.04
			
**CDK6**	0.95–1.39	0.73–1.06	0.94–1.33
			
**CDC20**	0.90–0.99	0.87–1.00	0.97–1.03
			
**CDC25B**	0.96–1.04	0.90–1.01	0.96–1.06
			

Notes. Using the 2^−ΔΔC_T_^ method [20], the data are presented as the range of fold change in gene expression relative to the unexposed controls at a given time point in NHEK cells.

## Results

### Effect of ELF-EMFs on cell growth and colony formation of the NHEK and HaCaT cells

**Figure 1:**
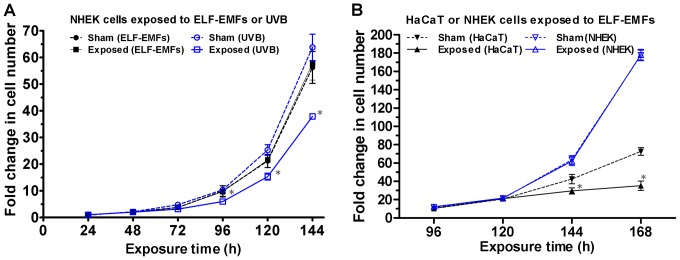
Effect of ELF-EMF exposure on cell growth of the NHEK and HaCaT cells. (A) NHEK cell growth curves for the sham (ELF-EMFs; •) and exposed (ELF-EMFs; ▪) groups for ELF-EMFs experiments; sham (UVB; blue ○) and exposed (UVB; blue □) groups for the positive controls (cells collected and counted during 24–144 h after being subjected to 21.5 J/m^2^ of UVB irradiation). The NHEK cells were seeded and incubated for 24 h prior to ELF-EMF exposure. The results are presented as the mean ± SD fold change in the cell count relative to the initial seeding cell count of 4 independent experiments (ELF-EMFs) or in triplicate (UVB; positive controls). Student's *t*-tests were conducted to analyze the differences between each sham and exposed groups. * *P*<0.05. (B) HaCaT cell growth curves for the sham (▾) and exposed (▴) groups for ELF-EMFs experiments; NHEK cell growth curves for the sham (blue ▽) and exposed (blue △) groups for ELF-EMFs experiments. A simultaneous exposure experiment revealed that the exposed HaCaT cells exhibited a lower cell number than the sham-exposed cells did after 144 h and 168 of ELF-EMF exposure, whereas no effects of ELF-EMFs on cell number were observed in NHEK cells. The results are presented as the mean ± SD fold change in the cell count relative to the initial seeding cell count of 3 independent experiments. Student's *t*-tests were conducted to analyze the differences between each sham and exposed groups. * *P*<0.05.

**Figure 2:**
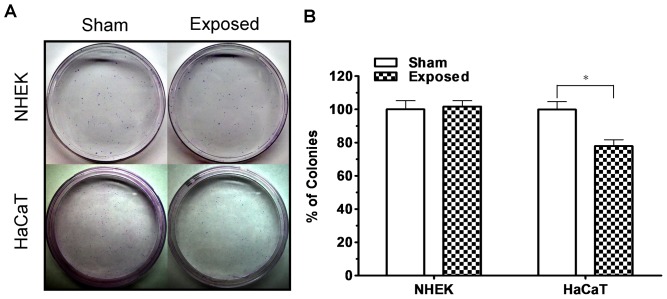
Effect of ELF-EMF exposure on colony formation of the NHEK and HaCaT cells. (A) Photograph of colony formation of NHEK cells (upper) and HaCaT (lower) cells after 144 h of ELF-EMF exposure. (B) The bars indicate the colony formation of the sham and exposed NHEK (left) and HaCaT cells (right) after 144 h of ELF-EMF exposure. The results indicated no influence of ELF-EMFs on cell proliferation efficiency of NHEK cells. The exposed HaCaT cells exhibited lower (77.96%±3.77% of sham-exposed cells) colony formation than did the sham-exposed cells. The results are presented as the mean ± SD of 3 independent experiments. A Student's *t*-test was conducted to analyze the differences between the sham and exposed groups. * *P*<0.05.

We previously demonstrated that ELF-EMFs can inhibit cell growth and reduce cell proliferation efficiency in HaCaT cells [13]. In the present study, we investigated the effect of ELF-EMFs on cell growth of NHEK cells. As shown in Figure 1A, a Student's *t*-test on the data revealed no significant differences in cell growth between the sham and exposed groups following ELF-EMF exposure. Also depicted in Figure 1A, the positive control of the UVB-irradiated cells began to exhibit significant decrease in the cell count at 72 h after being subjected to 21.5 J/m^2^ of UVB irradiation. To confirm that the 2 epidermal keratinocytes responded to ELF-EMFs differently, we simultaneously exposed the NHEK and HaCaT cells to ELF-EMFs in the same exposure area and extended exposure time to 168 h. The sham groups of both cell lines were simultaneously cultured in the same incubator and shielded from ELF-EMFs by the mu-metal box. The simultaneous exposure of the two cell types results indicated that the NHEK and HaCaT cells exhibited distinct responses to ELF-EMFs. As indicated in Figure 1B, the exposed cells exhibited a lower cell count than the sham-exposed cells did after 144 h of ELF-EMF exposure in HaCaT cells. This HaCaT cell growth result was consistent with our previous study [13]. However, ELF-EMFs exerted no effect on cell growth of NHEK cells. In addition to cell growth, we performed colony formation assays of NHEK cells and HaCaT cells to study cell proliferation efficiency after 144 h of ELF-EMF exposure. Figure 2A and Figure 2B indicated that ELF-EMFs exerted no influence on cell proliferation efficiency of NHEK cells. Nevertheless, the exposed HaCaT cells exhibited decreased (77.96%±3.77% of sham-exposed cells) colony formation compared with the sham-exposed cells. Our results indicated that ELF-EMFs exerted no effects on proliferation of NHEK cells but could reduce that of HaCaT cells.

### ELF-EMFs did not affect cell cycle distribution of NHEK cells

**Figure 3:**
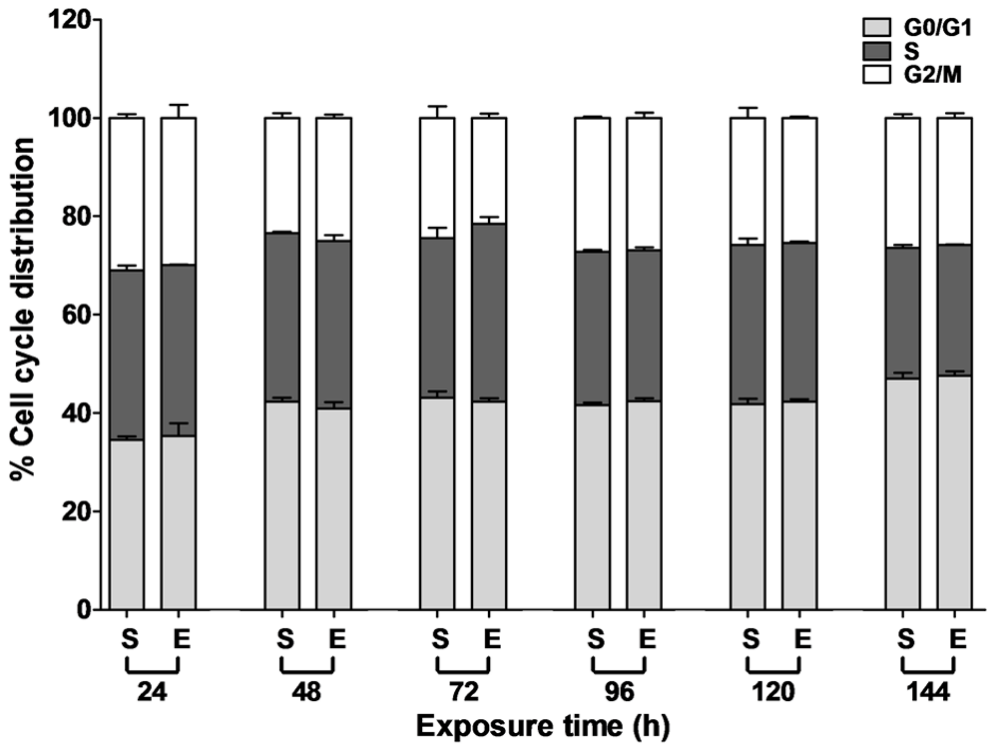
Effect of ELF-EMF exposure on cell cycle distribution of NHEK cells. NHEK cells were exposed to ELF-EMFs for 24 to 144 h and analyzed using PI staining and flow cytometry. The percentage of the cell cycle growth phases in the sham (S) and exposed (E) groups are presented as the mean ± SD of 3 independent experiments. A Student's *t*-test of the data revealed no significant difference in cell cycle distribution between the sham and exposed cells.

As reported in the previous study [13], ELF-EMFs caused G1 arrest in HaCaT cells. To investigate whether ELF-EMFs disturbed cell cycle progression of NHEK cells, the cell cycle distribution between 24 and 144 h of ELF-EMF exposure was analyzed by conducting PI staining and flow cytometry. Figure 3 indicated that ELF-EMFs did not alter cell cycle distribution of NHEK cells. The cell cycle distribution of NHEK cells differed from that observed in HaCaT cells after ELF-EMF exposure.

### The expression of cell cycle-related genes in NHEK cells after ELF-EMF exposure

As reported in the previous study [13] that through the cDNA microarray screening, 6 cell cycle-related genes (*CDKN1A*, *CCNA2*, *CCNB1*, *CDK1*, *CDC20* and *CDC25B*) are significantly differentially regulated in HaCaT cells after ELF-EMF exposure. In this study, we performed qRT-PCR analysis for 12 cell cycle-related genes (including the above mentioned 6 genes and *GADD45A*, *CCND2*, *CCNE1*, *CDK2*, *CDK4* and *CDK6*) of NHEK cells after 24–144 h of ELF-EMF exposure. Figure S1 showed that no significant difference existed in the gene expression levels of 12 cell cycle-related genes between the sham and exposed groups. Table 1 summarized the relative gene expression levels of the 12 cell cycle-related genes of the exposed NHEK cells relative to that of the unexposed controls. The qRT-PCR results indicated that ELF-EMFs exert distinct effects on the transcript level of cell cycle-related genes in the NHEK and HaCaT cells.

### ELF-EMFs did not activate ATM signaling cascades in NHEK cells

**Figure 4:**
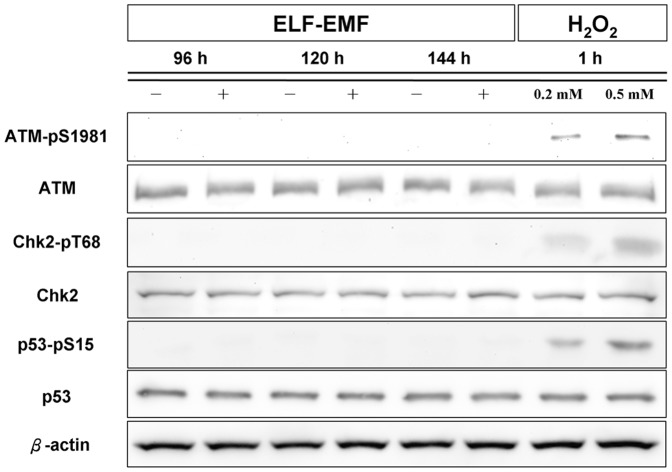
Immunoblotting of phospho-ATM (Ser1981), phospho-Chk2 (Thr68), and phospho-p53 (Ser15) after ELF-EMF exposure in NHEK cells. The expression levels of phospho-ATM (Ser1981), phospho-Chk2 (Thr68), and phospho-p53 (Ser15) were showed at the indicated times after ELF-EMF exposure in NHEK cells. β-actin was used as a loading control. Cells treated for 1 h with 0.2 mM or 0.5 mM H_2_O_2_ were used as positive controls in the experiments. All proteins were determined in whole cell lysates from the sham and exposed NHEK cells after the indicated exposure times. The blots shown are representative of three independent experiments.

We previously showed that ELF-EMFs cause inhibition of HaCaT cell growth through the activation of ATM/Chk2-dependent signaling cascades [13]. To investigate whether ELF-EMFs can activate ATM signal and other downstream signaling cascades of ATM, we performed immunoblotting assay to investigate the expression of the proteins involved in the ATM signaling cascades. Figure 4 showed that phosphorylation of ATM, phosphorylation of Chk2, and phosphorylation of p53 were not induced after 96 to 144 h of ELF-EMF exposure.

## Discussion

Previous studies have indicated that ELF-EMFs could cause genotoxic effects (DNA strand breaks or chromosomal damages) [21]–[23] which are cell type specific responses [11]. However, Scarfi et al. [24] could not reproduce these findings using comet and micronucleus assays; Burdak-Rothkamm et al. [25] also suggested that there is no significant effect for γH2AX assay, comet assays and chromosomal aberration induction in human fibroblasts exposure to ELF-EMFs. In 2010, Focke et al. [26] indicated that ELF-EMFs could induce increasing in comet tail DNA. Hence, there has been much debate and controversial studies regarding biological effects of ELF-EMFs. Although some of these studies proposed that ELF-EMFs may cause genotoxic effects (e.g. DNA strand breaks), the mechanism for DNA damage under ELF-EMF exposure is unclear. Because the ATM signaling pathway is involved in the DNA damage response [27]–[29], the study of ATM signaling pathway may be a suitable endpoint for investigating biological effects of ELF-EMFs.

Trillo et al. [4] reported significant differences in cell proliferation between the response of 2 human cancer cell lines to the combined treatment with all-trans-retinol (ROL) and ELF-EMFs. Moreover, some reports have indicated that contradictory results regarding the biological effects of ELF-EMFs may be caused by the viability of exposure systems, exposure conditions, and cell types [12], [30]. From our previous study [13], based on a simultaneous exposure methodology and rigorous exposure conditions, it presents results confirming the biological effects of HaCaT cells following exposure to ELF-EMFs. This study revealed that up to 144 h of ELF-EMF exposure did not affect cell growth, cell proliferation, or cell cycle distribution of NHEK cells. Moreover, the qRT-PCR data revealed that NHEK and HaCaT cells responded to ELF-EMFs differently in transcriptional level. Even though the NHEK and HaCaT cells are both epidermal keratinocytes, they respond differently to exogenous stresses [31]. In addition, the expression of several distinct proteins such as p53, p63, p21 was strikingly different in HaCaT and NHEK cultures at baseline [32]. Previous reports have indicated that exposure of HaCaT cells to UVB induces a significantly higher level of apoptosis compared to that in NHEK cells [33]–[36]. In contrast, other studies have demonstrated that NHEK cells are more radiosensitive than HaCaT cells to ionizing radiation (γ-irradiation) [37], [38]. These distinct responses to UVB and ionizing radiation between HaCaT and NHEK are suggested related to p53-dependent pathways [37], [39].

As depicted in Figure 1, the growth rate increased after 120 h exposure. We carefully performed at least three independent experiments and observed that NHEK cells exhibited a 1.44±0.11 (during 120–144 h) or 1.63±0.05 (during 144–168 h) times faster growth rate than they did in the early growth stages (before 120 h). We initially seeded lower cell density (165–750 cells/cm^2^) than that was recommended in the NHEK product sheet provided by ATCC [40] to allow cells to grow for at least 7 days without contact inhibition of cell growth, i.e. the culture approaching 80% confluence. According to our contact with ATCC, they have indicated that the lower seeding would grow more slowly and as the cells become greater in numbers they will be able to double at a faster rate until they reached the contact inhibition. A previous study of NHEK [41] has also reported similar situations. In this study, both the sham and exposed cells show the same growth character and we have ensure that they do not reach the contact inhibition.

In the case of HaCaT cells (p53-mutated), ELF-EMFs induce the activation of ATM signaling pathway resulting in G1 arrest [13]. The protein kinase ataxia-telangiectasia mutated, ATM, is an apical activator of DNA damage response (DDR) cascade [42], [43] and the activated ATM could phosphorylate downstream targets of the signaling pathway, such as p53 and Chk2 [44]–[47]. It is natural to suspect that the activation of ATM-Chk2-p21 pathway observed in HaCaT cells after ELF-EMF exposure might be blocked by the wild-type p53 protein, a downstream target of ATM, in NHEK cells. However, our immunoblotting data (Figure 4) depicted that that ELF-EMFs did not activate ATM or p53 in NHEK cells. Therefore, responding to ELF-EMF exposure, the two epidermal keratinocytes have essential difference beyond the distinct function of p53 protein. It is interesting to note that the distinct responses to UVB and ionizing radiation between HaCaT and NHEK may involve in p53-dependent pathways as mentioned earlier. Although the fundamental reasons that 2 epidermal keratinocytes respond to ELF-EMFs differently are unclear, our results imply that HaCaT cells may be more susceptible to ELF-EMFs than NHEK cells are.

In summary, our results revealed that the effects of ELF-EMF exposure on cell growth, cell proliferation, and cell cycle distribution differ among keratinocyte types. In other words, ELF-EMFs cause cell type-specific responses even among the keratinocytes. Our findings may partially explain the inconsistent results of previous studies when comparing results across various experimental models.

## Supporting Information

Figure S1 **The relative gene expression level of cell cycle-related genes in NHEK cells after ELF-EMF exposure.** The qRT-PCR was performed to determine (A) *GADD45A*, (B) *CDKN1A*, (C) *CCNA2*, (D) *CCNB1*, (E) *CCND2*, (F) *CCNE1*, (G) *CDK1*, (H) *CDK2*, (I) *CDK4*, (J) *CDK6*, (K) *CDC20* and (L) *CDC25B* gene expression levels in NHEK cells after 24–144 h of ELF-EMF exposure. The data are presented as the fold change in gene expression relative to the unexposed controls. The data are presented as the mean ± SD of triplicate. A Student's *t*-test of the data revealed no significant difference in the relative gene expression levels of 12 genes between the sham and exposed cells.
(TIF)

Table S1 **Primer sequences for quantitative real-time PCR.**
(DOC)
