# Supplementary figures and images for: Distinct Epidermal Keratinocytes Respond to Extremely Low-Frequency Electromagnetic Fields Differently
Source: PLoS One. 2014 Nov 19;9(11):e113424. doi: 10.1371/journal.pone.0113424 (PMC4237442; doi:10.1371/journal.pone.0113424)

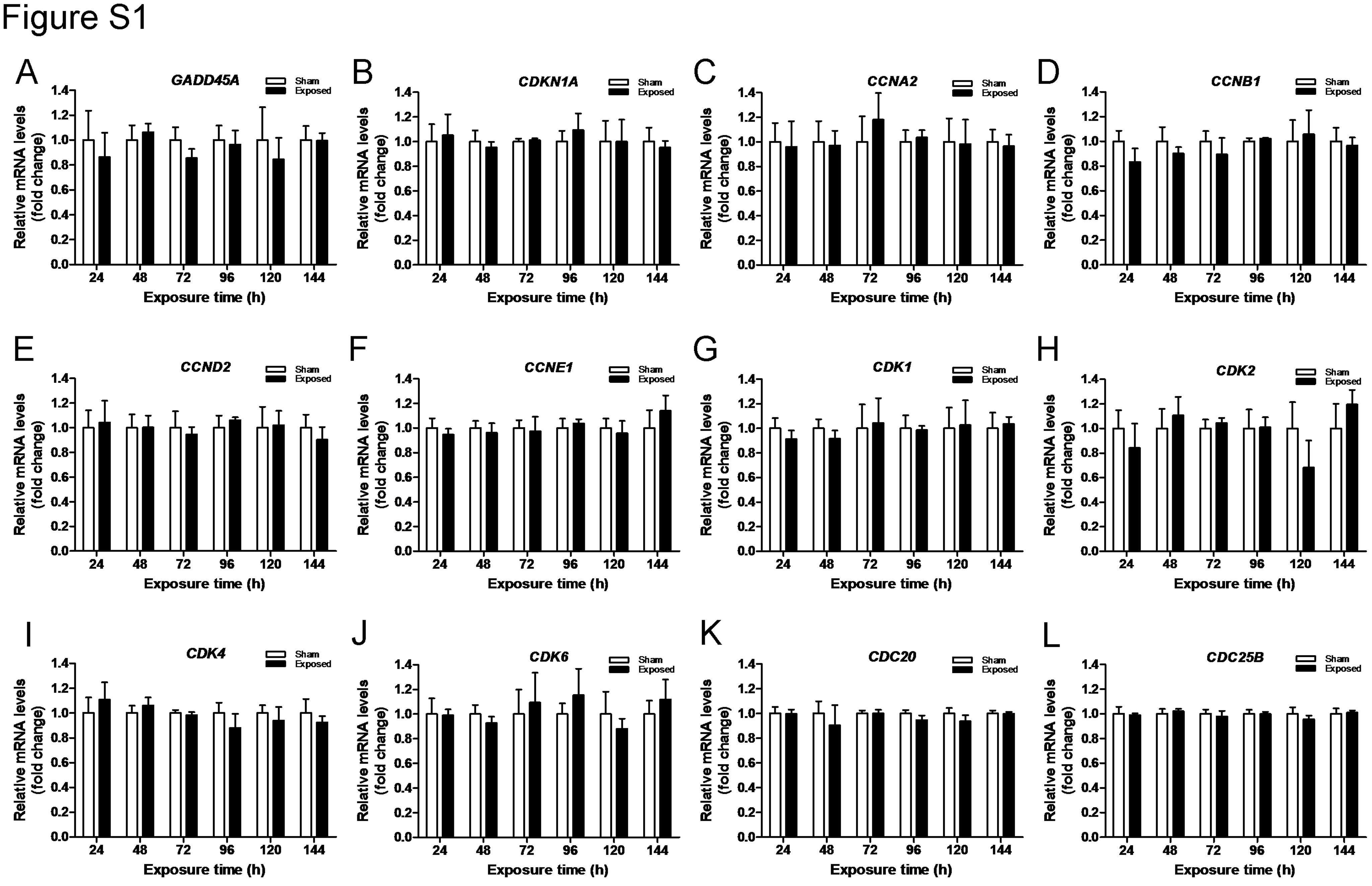

Supplement: Figure S1 — The relative gene expression level of cell cycle-related genes in NHEK cells after ELF-EMF exposure. The qRT-PCR was performed to determine (A) GADD45A, (B) CDKN1A, (C) CCNA2, (D) CCNB1, (E) CCND2, (F) CCNE1, (G) CDK1, (H) CDK2, (I) CDK4, (J) CDK6, (K) CDC20 and (L) CDC25B gene expression levels in NHEK cells after 24–144 h of ELF-EMF exposure. The data are presented as the fold change in gene expression relative to the unexposed controls. The data are presented as the mean ± SD of triplicate. A Student's t-test of the data revealed no significant difference in the relative gene expression levels of 12 genes between the sham and exposed cells. (TIF) [file pone.0113424.s001.tif]
