# Supplementary material for: Distinct Epidermal Keratinocytes Respond to Extremely Low-Frequency Electromagnetic Fields Differently
Source: PLoS One. 2014 Nov 19;9(11):e113424. doi: 10.1371/journal.pone.0113424 (PMC4237442; doi:10.1371/journal.pone.0113424)
Supplement: Table S1 — Primer sequences for quantitative real-time PCR. (DOC) [file pone.0113424.s002.doc]

**Table S1. Primer sequences for quantitative real-time PCR**

| **Gene Name** | **Entrez Gene ID** | **Forward primer sequence (5' to 3')** | **Reverse primer sequence (5' to 3')** |
| --- | --- | --- | --- |
| GADD45A | 1647 | TCTCCCTGAACGGTGATGGC | CAGCCCCTTGGCATCAGTTT |
| CDKN1A | 1026 | TTCTACCACTCCAAACGCCG | GCAGAAGATGTAGAGCGGGC |
| CCNA2 | 890 | GCACTGGTGGTCTGTGTTCTGT | CTTCTTGGATGCCAGTCTTACTCA |
| CCNB1 | 891 | TGACATGGTGCACTTTCCTCC | AGGTGCTGCATAACTGGAAGAAG |
| CCND2 | 894 | ATCGGGCTCCCAGTTAGACC | CGACATCTGAGCCTACCGC |
| CCNE1 | 898 | TCCTGGATGTTGACTGCCTTG | ATGTCGCACCACTGATACCCT |
| CDK1 | 983 | AGGATTTTCAGAGCTTTGGGC | ATGCTAGGCTTCCTGGTTTCC |
| CDK2 | 1017 | TTAAGTTAGCCTCCACCACCCT | GGGGCATTCAGAGATTGGTAC |
| CDK4 | 1019 | CTGTGCCACATCCCGAACT | TCCTTGATCGTTTCGGCTG |
| CDK6 | 1021 | CAAGGTGGTCAGTAAATAACAGGCA | AAGAAATGCTGAGGACATGGGG |
| CDC20 | 991 | GACCGCTATATCCCCCATCG | TTCCTTCTTGGTGGGCGTCT |
| CDC25B | 994 | CTGCCATGTTGCCCCTTTCT | GAAGTGTCCTGAGATGGGCGT |
| GAPDH | 2597 | GGGGAGCCAAAAGGGTCAT | CCAGGGGTGCTAAGCAGTTG |
